# Response: Commentary: “Clinical evaluation of pediatric olfactory disorders: a review from etiology to management”

**DOI:** 10.3389/falgy.2026.1939998

**Published:** 2026-08-24

**Authors:** Eleonora M. C. Trecca, Manuel Marra, Marella Reale, Luca Leone, Michele Cassano, Antonio della Volpe, Ignazio La Mantia, Fabio Pagella, Elena Cantone

**Affiliations:** 1 Unit of Otolaryngology-Head and Neck Surgery, Department of Clinical and Experimental Medicine, University of Foggia, Foggia, Italy; 2 Otorhinolaryngology Unit, Head and Neck Department, Meyer Children’s Hospital IRCCS, Florence, Italy; 3 Otolaryngology Department, Santobono-Posillipon Hospital, Naples, Italy; 4 Faculty of Medicine and Surgery, University of Enna ‘’Kore’’, Enna, Italy; 5 Department of Surgical Science, University of Pavia, Pavia, Italy; 6 Department of Otorhinolaryngology, Fondazione IRCCS Policlinico San Matteo, Pavia, Italy; 7 Department of Pharmacy, Health and Nutrition Sciences, University of Calabria (UNICAL), Cosenza, Italy; 8 Otolaryngology Unit, Annunziata Hospital, Cosenza, Italy

**Keywords:** adenoid hypertrophy, hyposmia, Kallman syndrome, olfactory assessment, olfactory training, otolaryngology, pediatric olfactory disorder

## Introduction

We are grateful to Spencer and Bhargava for their careful commentary on our review and for identifying children in whom olfactory dysfunction is particularly likely to be missed: those with congenital, syndromic, neurodevelopmental or craniofacial conditions (1–4). These children may neither report smell loss nor reliably complete conventional psychophysical tests, highlighting an important gap in paediatric olfactory care (1, 5–8).

We interpret their proposal not as a separate modification of the original diagnostic pathway, but as a refinement within a broader clinical model guided by risk-based case finding, feasibility-based testing and treatment-oriented classification.

## From symptom-driven to risk-driven screening

Spencer and Bhargava correctly point out that the absence of a smell complaint should not reassure clinicians when a child belongs to a high-risk group (1). The evidence they cite—including congenital causes accounting for roughly two-thirds of paediatric olfactory dysfunction and impairment being detected in over 80% of children with CHARGE syndrome when all assessment modalities are considered—supports systematic, risk-triggered assessment (1, 4). In CHARGE, Kallmann syndrome, 22q11.2 deletion syndrome and Bardet–Biedl syndrome, olfactory dysfunction may be overlooked while attention is directed toward hearing, vision, endocrine, airway, feeding or developmental issues (1, 2, 9–11).

This strengthens the first step of the original diagnostic pathway (2). Congenital or syndromic diagnoses should not be recorded merely as background information; in selected children, they should actively trigger olfactory enquiry, safety counselling and, when appropriate, multidisciplinary assessment. Their observation further suggests that, where olfactory dysfunction is highly prevalent within a syndrome, its role may eventually warrant consideration in revised diagnostic criteria. Such a change, however, requires future consensus rather than incorporation into an individual clinical algorithm (1).

## Feasibility-based assessment

Conventional psychophysical tests have important limitations in some children (5–8). The proposed operational criteria for a “developmental bypass” pathway — non-verbal developmental age below approximately 4 years, inability to follow a two-step command, uncorrected bilateral choanal atresia or prolonged enteral feeding without established oral intake — provide a useful starting point for clinical discussion (1). However, as the authors acknowledge, these pragmatic thresholds are not formally validated and require prospective evaluation before routine use.

The proposed bypass should not be restricted to congenital syndromes alone. Developmental age, language comprehension, attention span, behavioural cooperation, nasal airflow and feeding route may all influence test validity (5–8). A child with autism spectrum disorder, severe attention difficulties or marked nasal obstruction may produce unreliable results even without a classic congenital syndrome. Conversely, some older children with syndromic conditions may complete adapted psychophysical testing successfully. The relevant distinction should therefore be between testing conditions that can provide reliable information and those that cannot.

When psychophysical testing is feasible, age-appropriate and culturally adapted tools remain central to diagnosis and follow-up (6–8). Otherwise, clinicians should combine structured history, feeding and safety assessment, nasal endoscopy, cautious parental report, selected imaging and, where available, objective electrophysiological investigations (1, 2, 5, 12). We use Spencer and Bhargava's term “developmental bridge” to denote this temporary alternative assessment pathway when reliable psychophysical testing is not yet possible, coupled with planned reassessment as the child's developmental or communicative abilities improve (1).

## Objective methods: potential and limitations

Magnetic resonance imaging is valuable in selected patients, particularly when congenital or central olfactory dysfunction is suspected (2). It may identify olfactory bulb or tract hypoplasia/aplasia, anterior skull base abnormalities, forebrain malformations or other lesions relevant to etiological classification. Nonetheless, imaging should not substitute functional assessment, since morphology and function may not always overlap (13).

Olfactory event-related potentials (OERPs) are among the most promising objective tools for children who cannot provide reliable behavioural responses (12). The commentators appropriately call for OERP technology to progress from a research tool toward routine clinical practice. Achieving this transition will require multicentre standardization of stimulation and recording protocols, paediatric normative data and clinically meaningful thresholds (1, 5, 12). Until these requirements are met, clinical translation will remain limited.

## Preserving the treatable dimension of paediatric olfactory dysfunction

While congenital and syndromic olfactory dysfunctions are diagnostically vulnerable, inflammatory and obstructive causes remain relevant because they may be modifiable (2–4). Adenoid hypertrophy, allergic rhinitis, chronic rhinosinusitis, nasal polyposis, cystic fibrosis-related sinonasal disease and post-infectious olfactory dysfunction should be actively identified, since medical, surgical or rehabilitative interventions may improve nasal patency, inflammation, food enjoyment and quality of life (2, 14, 15).

Refinement of the congenital arm should therefore not narrow the diagnostic perspective. A paediatric algorithm should identify hidden congenital dysfunction while preserving attention to reversible causes. Retronasal olfaction, appetite, feeding behaviour and family observations may be particularly informative in children with chronic nasal obstruction or inflammatory airway disease, although standardized paediatric assessment remains limited (2, 14, 15).

## Discussion

Spencer and Bhargava's proposals can be integrated into a risk–feasibility–treatability model. Risk-based triggers should prompt evaluation even without a spontaneous complaint (1, 9–11), while feasibility should determine whether psychophysical testing is reliable or alternative tools are needed (5–8). Finally, classification should distinguish congenital or sensorineural conditions, for which counselling, safety measures and follow-up are central, from inflammatory, obstructive, infectious or traumatic conditions in which treatment may modify outcomes (2, 14, 15).

**Figure 1:**
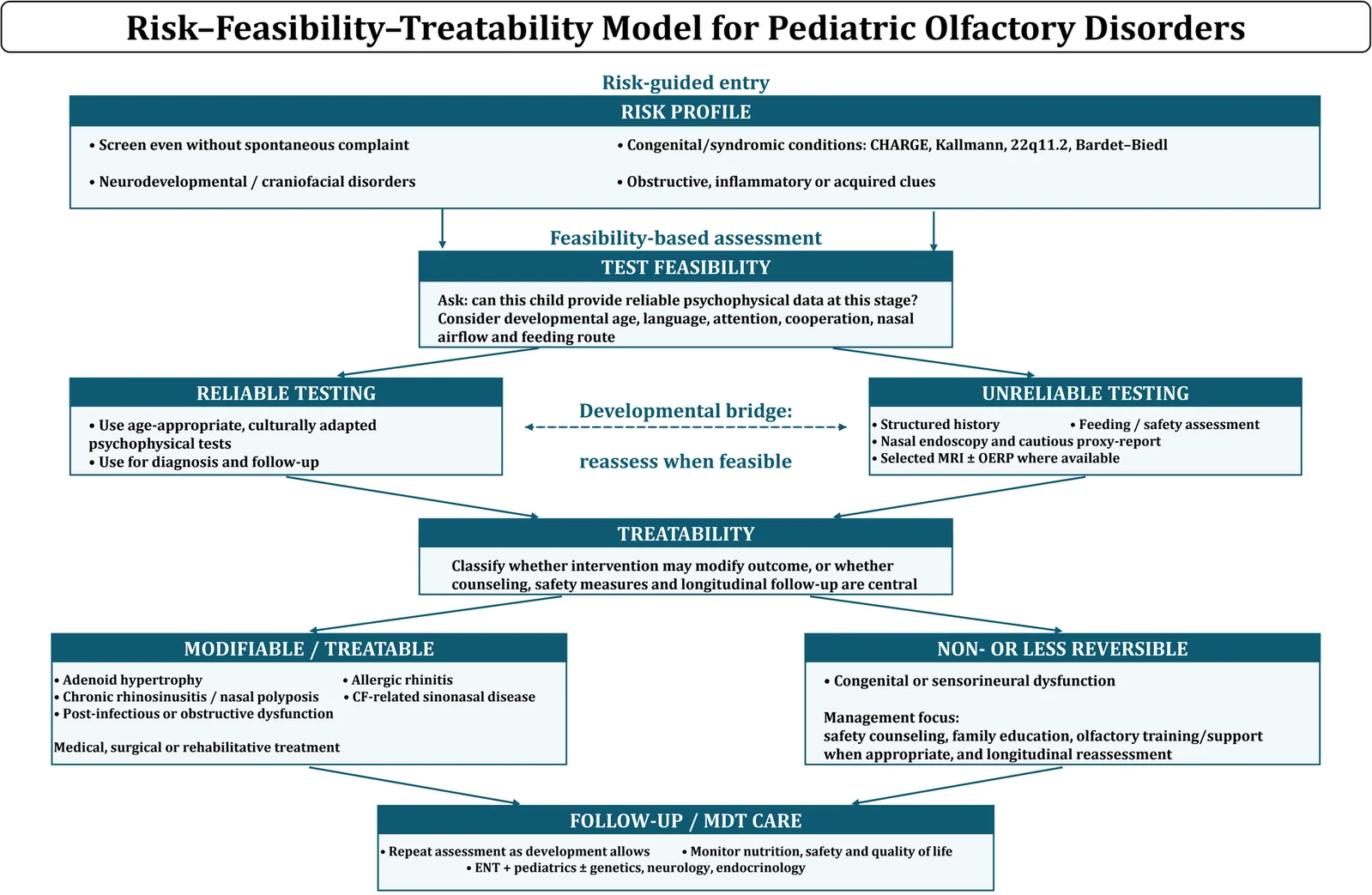
Proposed integration of the original paediatric olfactory disorder pathway with risk-triggered screening, feasibility-based assessment and treatment-oriented classification. This algorithm is a conceptual clinical framework and requires prospective validation before widespread implementation. CF, cystic fibrosis; CHARGE, coloboma, heart defects, choanal atresia, retardation of growth and development, genital abnormalities, and ear abnormalities; ENT, ear, nose and throat; MDT, multidisciplinary team; MRI, magnetic resonance imaging; OERP, olfactory event-related potentials.

The proposed integration is summarized in Figure 1. This model maintains the pragmatic structure of the original algorithm while accounting for developmental constraints. Future progress will require multicentre validation of age-adapted psychophysical tests, standardization of paediatric OERP protocols, longitudinal evaluation of syndromic populations and stronger incorporation of olfactory assessment into multidisciplinary paediatric care (3, 5, 6, 12).

In conclusion, we welcome Spencer and Bhargava's commentary as an important contribution to diagnostic equity in paediatric olfactory dysfunction. Their proposal should not replace the existing clinical pathway but enrich it by making explicit a principle central to paediatric practice: assessment should follow the child's risk profile, developmental abilities and potential for therapeutic benefit.
